# Processing Methodologies of Wet Microalga Biomass Toward Oil Separation: An Overview

**DOI:** 10.3390/molecules26030641

**Published:** 2021-01-26

**Authors:** Vânia Pôjo, Tânia Tavares, Francisco Xavier Malcata

**Affiliations:** 1 LEPABE—Laboratory for Process Engineering, Environment, Biotechnology and Energy, Faculty of Engineering, University of Porto, Rua Dr. Roberto Frias, 4200-465 Porto, Portugal; vpojo@fe.up.pt (V.P.); fmalcata@fe.up.pt (F.X.M.); 2 FEUP—Faculty of Engineering, University of Porto, Rua Dr Roberto Frias, 4200-264 Porto, Portugal

**Keywords:** microalgae, lipids, PUFA, wet biomass, pretreatment, extraction, biorefinery

## Abstract

One of the main goals of Mankind is to ensure food system sustainability—including management of land, soil, water, and biodiversity. Microalgae accordingly appear as an innovative and scalable alternative source in view of the richness of their chemical profiles. In what concerns lipids in particular, microalgae can synthesize and accumulate significant amounts of fatty acids, a great fraction of which are polyunsaturated; this makes them excellent candidates within the framework of production and exploitation of lipids by various industrial and health sectors, either as bulk products or fine chemicals. Conventional lipid extraction methodologies require previous dehydration of microalgal biomass, which hampers economic feasibility due to the high energy demands thereof. Therefore, extraction of lipids directly from wet biomass would be a plus in this endeavor. Supporting processes and methodologies are still limited, and most approaches are empirical in nature—so a deeper mechanistic elucidation is a must, in order to facilitate rational optimization of the extraction processes. Besides circumventing the current high energy demands by dehydration, an ideal extraction method should be selective, sustainable, efficient, harmless, and feasible for upscale to industrial level. This review presents and discusses several pretreatments incurred in lipid extraction from wet microalga biomass, namely recent developments and integrated processes. Unfortunately, most such developments have been proven at bench-scale only—so demonstration in large facilities is still needed to confirm whether they can turn into competitive alternatives.

## 1. Introduction

The increasing importance of following and maintaining a healthy diet is apparent; however, most people are unwilling to do so unless drastic changes in their living habits can be avoided. The need for novel substances to prevent health conditions related to high caloric diets, combined with sedentary lifestyles has accordingly been growing. Chronic diseases, such as heart disease, stroke, cancer, chronic respiratory diseases, and diabetes, are currently the leading causes of mortality in the world—and already represent 60% of all deaths. This invisible epidemic hinders economic development of many countries. The reduction in incidence and the treatment of chronic diseases using dietary supplements is expected to promote health at large and reduce healthcare costs. Hence, consumers have gradually evolved from a traditional understanding of “food” as a leading source of nutrients to a much more demanding quest that includes a potential to improve health.

Considering population growth, ocean overfishing, and limited resources of arable land and fresh water, there is a need for alternative natural nutrients from sustainable and scalable options—and containing bioactive compounds, able to prevent and/or treat diseases/conditions. Microalgae have, in that particular, earned the label of sources of potentially “bioactive” products.

Microalgae have been widely explored because of the diverse and useful applications of their (added-value) metabolites—of prime importance in the production of cosmetics, pharmaceuticals, food additives, animal feed (including aquaculture), vitamins, pigments, and biofuels [1,2,3,4,5]; they are indeed considered as innovative contributions to adequate nutrition [6]. Microalgae contain a valuable amount of organic compounds, such as proteins (40–70%), lipids (8–70%), carbohydrates (11–56%), and pigments (3–5%) [7,8]—responsible not only for their nutritional value, but also serving as sources of (or leads for) bioactive compounds [6,9]. In the last few decades, industrial cultivation of microalgae has increased dramatically [10]—and the corresponding market already generates an income of ca. US$720 million per year. Current production amounts to a total of 10 million tons of dry biomass of *Arthrospira* (spirulina), *Chlorella*, *Haematococcus*, *Euglena*, and *Dunaliella* (of which 80% is located in China); however, the production cost of microalgae biomass is still too high for widespread commercial applications [11,12].

One of the most competitive advantages of using microalgae is that they do not compete with traditional food crops for space and resources, and possess numerous economic and environmental benefits [13]. Compared with other crops, they prove more efficiency in solar energy capture; rapid growth (doubling time of ca. 24 h); performance essentially independent of weather changes; and drastically lower irrigation water needs. Furthermore, they exhibit a high capacity for CO_2_ sequestration (183 tons of CO_2_ /100 tons of microalgal biomass) [14] and wastewater treatment. Last but not least, microalgal lipid content can reach 25 to 200-fold that found in higher plants [15].

### 1.1. Lipids and Fatty Acids

Microalgae accumulate a diverse portfolio of lipids, which vary from species to species. Their lipid profiles also vary with cultivation conditions, such as medium composition, illumination intensity, temperature, and aeration rate [16,17,18,19]. The main lipid classes found in microalgae are acylglycerides, phospholipids, glycolipids, lipoproteins, free fatty acids, sterols, hydrocarbons, and pigments. These appear in distinct cellular locations, perform different cellular functions, and exhibit disparate polarity, viscosity, and solubility. Triacylglycerides (TAGs) and sterol esters bounded by a phospholipidic monolayer are present in the cytoplasm as a form of energy storage [20]. Diacylglycerol (DAG) and monoacylglycerol (MAG) are critical intermediates in TAG biosynthesis or enzymatic hydrolysis. Free fatty acids (FFAs) do not directly participate in cell metabolism, unless bound to other molecules.

Polyunsaturated fatty acids (PUFAs), namely omega-3 and omega-6, are essential components of the human diet [21,22,23]; those fatty acids, like essential amino acids, cannot be synthesized by the human body. Therefore, the intake from external sources is imperative. In the past decades, the most important of those compounds have been docosahexaenoic acid (DHA, 22:6, *n*-3), eicosapentaenoic acid (EPA, 20:5 *n*-3), α-linolenic acid (ALA, 18:3 *n*-3), arachidonic acid (ARA, 20:4 *n*-6), and γ-linolenic acid (GLA, 18:3, *n*-6); they have attracted considerable interest in what concerns human health, namely for modulating the risk of prevalent diseases [24,25,26]. Those long-chain PUFAs (LC-PUFAs) (namely DHA and EPA) play a favorable role, particularly in reducing such cardiovascular complications as arrhythmia, stroke, and high blood pressure [3,4]. Additionally, they offer beneficial effects upon depression, rheumatoid arthritis and asthma [27,28,29], inflammatory bowel disease, and some forms of cancer [21,29,30,31,32].

The traditional DHA and EPA source in human consumption is cold-water fish species, including salmon, mackerel, mullet, and herring [23,33,34]. Mass-scale fishing is no longer sustainable as fisheries already use up the maximum fish stocks per annum, to supply the market with fish for human consumption, feed for industrial fish farms, and raw materials for oil supplements. Therefore, the need to protect fish species and marine ecosystems, coupled with the need to prevent chronic diseases worldwide have led to intensive search for alternative sources of LC-PUFAs [35]. Among the various strategies proposed—including bacteria, fungi, plants, and microalgae [36,37,38,39,40,41], microalgae-based lipids have been found the best alternative to provide healthy fatty acids for human diets [42]. In addition, the drawbacks associated with use of using fish-oil—such as chemical contaminants [43,44], unsuitability for vegetarians, unsustainable supply, and presence of off-odors that make fish oil unattractive, can be easily overcome [45].

Microalgae, on the other hand, are the original DHA and EPA producers in the marine food chain. They grow fast under various autotrophic, mixotrophic, and heterotrophic culture conditions, and exhibit a high LC-PUFA production potential [46]. Consequently, several microalgae species are promising sources—and probably the only economically feasible alternative to fish oils when seeking for DHA and EPA [47]. Many microalga species can accumulate lipids to very high levels indeed; for instance, *Auxenochlorella protothecoides* goes up to 70% of its dry biomass as lipids [48,49]. *Pavlova lutheri* produces PUFAs at large scale, and *Phaeodactylum tricornutum* can accumulate EPA up to 30–40% of their total fatty acid inventory; while *Schizochytrium* sp. can accumulate ca. 50% of its total lipids as DHA [22,50].

### 1.2. Lipids Recovered

Despite the advantages associated with use of microalgae as raw material, the lipid recovery process poses several limitations—which must be overcome, in order to make it commercially competitive. Typically, five-step process is used for microalgae: cultivation, harvest, drying, lipid extraction and fractionation.

Presently the dominant constraint in sustainable oil production from microalgae is the efficient and safe extraction of lipids, which correlates with high downstream processing costs. Methodologies for lipid extraction from dried biomass have been reported to be more efficient—and are thus the most commonly elected in practice; examples include Folch [51], Bligh and Dyer [52], and Soxhlet [53] methods. However, they are time-consuming (may even take up to 3 days until conclusion) and contaminate the residual biomass with solvent residues—thus compromising its subsequent application for other purposes. Moreover, drying the biomass represents 89% of the whole energy input [54], and 70–75% of the total processing cost [55]—and may concomitantly alter the structure of lipids. Therefore, it has become clear that the drying step dramatically limits the overall process efficiency, and makes it hardly suitable for large-scale operation [56,57,58,59,60,61,62]. To remove the problems associated with drying, lipid extraction from wet biomass has emerged as a potential alternative [63]. Dong et al. [64] suggested a promising approach for the release of FFAs from their bound moieties before or after extraction, with the likely removal of unwanted impurities. This may constitute a possible solution to the economic feasibility of large-scale production of bio-oil from microalgae.

Lipid recovery from wet biomass is affected by several factors, e.g., lipid accessibility, mass transfer limitations, emulsification, and presence of insoluble biomass residues [64]. Recall that microalgae possess thick and robust cell walls, responsible for their mechanical resistance—which need to be disrupted upon harvest and before release of oil [63,65,66]. Moreover, the polar lipid hydrophilic portions complicate extraction with non-polar lipids (TAGs and FFAs), and impair wet extraction because they can act as emulsifiers [64]. To properly extract lipids from wet microalga biomass, biomass pretreatment is necessary to disrupt the cells. This pretreatment must guarantee opening of the cell wall, exposure of the cytoplasmic lipids, decrease in mass transfer limitations, and reduction in extent of emulsion formation. Novel wet harvesting strategies to extract major lipids have been claimed to exhibit a cost-efficiency 30% less than conventional strategies [67]; hence, work is still required toward development of new energy- and cost-efficient, and environment-friendly methods suitable for application on a large scale.

On the other hand, industrial facilities dealing with microalgae are usually composed of stand-alone or minimally integrated configurations—where raw materials, energy expenditure, and product delivery act separately, thus increasing overall operational expenses [68]. For such a process to develop into a competitive reality, it should integrate microalga production with new or existing large-scale facilities. The concept of process integration has gained importance in recent times, toward recovery of several compounds from biomass or raw materials available at adjacent industrial units [69,70]. Therefore, R&D efforts are needed at the industrial level to optimize integrated production—provided that they will reduce the usage of energy and materials throughout the whole chain, from cultivation to end-use [71]. Such innovative technological routes should also comply with green engineering requirements.

### 1.3. Microalgae Cell Wall

By definition, lipid extraction from wet algal biomass consists of disrupting/damaging the cell walls while suspended in the cultivation medium; remember that the said cell wall provides a robust excellent protection against the environmental medium.

**Figure 1:**
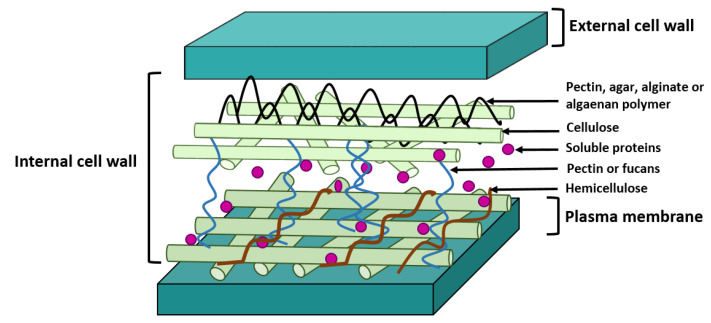
Composition of typical microalgal cell wall (redrawn from [72]).

Most microalgal cell walls comprise polysaccharides (i.e., cellulose, hemicellulose, pectin, alginates and/or algaenan), and proteins (i.e., glycoproteins) [72] (Figure 1). However, the composition is species-dependent—including distinct chemical structures and relative proportions; this justifies why an unequivocal microalgal cell wall structure cannot be strictly established. Cell wall may range from tiny a membrane to a multilayered-complex structure [73,74]. It is generally accepted that green microalgae (e.g., *Scenedesmus*, *Chlorella*, *Nannochloropsis*) possess a more rigid cell wall than red algae or cyanobacteria [75]; the former encompass indeed an algaenan-based layer—a nonhydrolyzed polymer that accounts for the difficulty in disrupting of microalgal cell walls [76]. One exception worthy of mention is the genus *Dunaliella*—which differs from the other green microalgae because it lacks a polysaccharide-based cell wall, being instead surrounded by numerous mucilage layers [77,78].

The efficiency of the disruption technique (and thus its choice among the various possibilities available) will accordingly be dependent on cell size, cell wall composition, and growth conditions. A few studies emphasized that a given set of disruption techniques and operating conditions produce different cell disruption efficiencies when applied to distinct microalga species [64]. Unfortunately, the hard and rigid cell walls of microalgae restrict use of selected components of their biomass at an industrial scale, owing to the difficulty in extracting intercellular components [79].

## 2. Biomass Pretreatments for Lipid Extraction Applied to Wet Biomass

Pretreatments can be applied to biomass to enhance efficiency in lipid recovery by breaking or weakening the microalgal cell walls, thus facilitating cellular lipid extraction [29,30]. Meanwhile, the biomass pretreatment process should be energy-efficient and scalable for industrial applications. Pretreatment methods applied to wet biomass will be critically reviewed below.

### 2.1. Microwaves

Microwave-assisted extraction induces the release of lipids from the cells by dielectrically heating the medium water (as a polar solvent), which generates steam inside the cells and induces changes in their structure that include breakage that leads to electroporation, thus opening up of cell membrane and allowing the release of intracellular compounds [80,81]. It has been proven an efficient, harmless, and fast technique when applied to wet microalga biomass [82]. Several researchers have explored the profitability of using this technology compared to alternatives, and accordingly tested different powers and exposure times. This is the case of the work by Biller et al. [83], who compared utilization of dichloromethane upon unprocessed and microwaved samples of *Nannochloropsis occulata, Chlorogloeopsis fritschii* and *Pseudochoricystis ellipsoidea*—using 1 g of freeze-dried microalgae with 10 mL of deionized water (to form a slurry); the samples were heated to 80, 100, 120, and 140 °C within 3 min. Those authors observed that microwave processing greatly improved lipid extraction from all strains, with 3–7-fold increases even at the lowest temperature. They also found no changes in lipid profile at the different temperatures; and claimed that this approach works better with marine algae. It removes substantial amounts of the inorganic ash fraction, thus allowing use of the biomass feedstock for additional processing; and the increased ionic conductance will require less energy for heating a posteriori.

Microwave treatment was also investigated by Cheng et al. [84]; they compared its impacts with those caused by conventional thermal heating, upon lipid extraction from wet *Chlorella pyrenoidosa* (with a water content of 80 wt.%). The following conditions were utilized: microwaves were used simultaneously with lipid extraction and transesterification (one-step method), or lipid transesterification with conventional thermal heating (two-step method). The authors reported the former to be more efficient, since it allowed rupture of ca. 77.5% of the wet microalgal cell walls.

Cheng et al. [85] discussed an innovative process for efficient disruption of wet microalgae, with subsequent transesterification of the released lipids. Microwaves were used to disrupt the cells, and transesterification took place after lipid extraction with hexane. The amount of FAME obtained via this process was 6-fold that by the other two processes investigated.

Wahidin et al. [86] compared the microwave method with conventional water bath-assisted solvent extraction—to ascertain which one would be more efficient to extract lipids from *Nannochloropsis* sp. They showed that microwaves lead to higher lipids content (38.3%), compared to the water bath-assisted solvent extraction method (23.0%, as (determined by gravimetry).

A study to ascertain the possibility of overcoming high-moisture problems in conversion processes was published by Bach et al. [87]; they checked the efficiency of wet torrefaction (WT) of *C. vulgaris* (20 g of dried microalga for 100 g of distilled water), using a microwave-assisted heating system. Their results revealed a significant impact of WT upon combustion reactivity of the microalga; the carbohydrate content was lowered, whereas the protein and lipid contents increased.

A significant improvement in FAMEs production from microalgal biomass can be achieved by combining two pretreatment techniques—microwave and ultrasound irradiation. Although microwave and ultrasound irradiation are well known for their individual efficiency, either one has several limitations which will be overcome if those two techniques are combined. For example, microwave raises mass transfer limitation, while ultrasound lacks the rapid heating capability of microwave. Therefore, integration of these technologies will likely enhance reaction rate, disruption of algal cells, and mass transfer [88]. Martinez-Guerra et al. [89] evaluated the associated synergistic effect upon *Nannochloropsis* sp., using response surface methodology encompassing five process variables (reactant and catalyst volume, reaction time, ultrasound power, and microwave power), toward maximum FAMEs production; the effect of those variables was studied via a central composite design (CCD). Reaction kinetics for wet microalgae transesterification reaction, at different reaction temperatures, was accordingly evaluated; it was found that microwave energy dissipation at a rate of 140 W, combined with 140 W of ultrasound intensity, was adequate to produce FAMEs to a maximum yield of 48.2%.

Another synergistic effect was found between a microwave-assisted system and polyvinyl chloride (PVC) to bring about co-pyrolysis of microalgae—as demonstrated by Dai et al. [90]. Microalgae has been acknowledged to be a potential alternative to fossil fuels; however, pyrolytic oil therefrom holds several disadvantages, such as low heating value, poor stability, and high acidity, owing to its high oxygen and low hydrogen contents [91]. To assess the relative hydrogen content of different samples, an effective hydrogen index (EHI) should be calculated. The EHI of microalgae lies usually below 0.3, thus indicating an extreme lack of hydrogen. Co-pyrolysis of microalgae with other materials bearing higher EHI would be a useful way to improve the physicochemical properties of the oil, as a result of hydrogen transfer and synthetic effects during co-pyrolysis. The authors analyzed the effect of mixing ratio on product yield at different microwave powers and reported synergisms between microalgae and PVC concerning bio-oil production. Maximum yields were indeed attained when microalgae to PVC ratios were 5:5 and 3:7 at 800 W, and 5:5 at 1000 W, respectively. Effective treatment of PVC is urged for environmental protection. Since PVC seems to be a suitable hydrogen donor (EHI = 1.64) and the pyrolytic residue obtained from microalgae may absorb the chlorine volatiles originated from PVC, combination thereof is of great significance in attempts to reach an efficient and low-pollution form of energy conversion.

Extraction of lipids assisted by the microwave technique brings several advantages to the process of biodiesel production—namely, the fact that it becomes smoother, preserves the integrity of extracted compounds, allows for simultaneous extraction of several bioproducts, and requires short reaction times.

Following a detailed analysis of recent publications related to application of microwave processing to industrial scale, it was found that the energy requirement for microwave irradiation can be as high as 420 MJ per kg of dried biomass [92,93]. This energy requirement hampers large-scale processing of microalgae. Additionally, most studies available have not focused on the relevance of this technique toward development of industrial prototypes beyond (routinely used) laboratory scale rigs—even though some of them have discussed scale-up issues [94,95,96,97,98,99,100,101,102]. The scarcity of knowledge from demonstration plants often prevents successful scale-up, because the associated technical risks have not yet been fully ascertained as needed for effective mitigation thereof [101].

### 2.2. Ultrasound

Ultrasound-mediated extraction breaks the cells through the process of cavitation (in a liquid environment) generated by the ultrasonic wave, as it gives rise to formation of microbubbles around the cells. These microbubbles collapse, and concomitantly create a shock wave—sufficient to break the cell wall and lead to intracellular component release [103,104]. This technique is fast, and high purity of the final product is reached. Sung and Han [105] investigated ultrasound-assisted, in-situ transesterification on wet *Aurantiochytrium* sp.—using potassium carbonate as alkaline catalyst, because it was previously found that water strongly decreases process efficiency. This method proved a viable technique to overcome mass transfer limitations associated with use of wet biomass, thus allowing a high yield of fatty acids be reached.

Ultrasound can be used in conjunction with other techniques to increase extraction yields, as done by Sankaran et al. [106]. They investigated a sugaring-out extraction technique supported by liquid biphasic flotation, coupled with ultrasonication for protein removal. Microscopic images of cell morphology of *C. vulgaris*, before and after ultrasonication, proved it to be a suitable method to damage cells—and thus promote release of their intracellular components.

When comparing ultrasound-assisted extraction with Bligh and Dyer’s conventional methodology, the latter was found more efficient in terms of lipid recovery. Adam et al. [100] reported a higher amount of FAMEs from *Nannochloropsis oculata* fresh biomass, extracted via Bligh and Dyer’s methodology. However, ultrasound-assisted extraction preserves the quality of fatty acids; and for being a green technology, it circumvents use of solvents while allowing oil recovery in a reduced timeframe.

The efficiency of ultrasound for cellular disruption was also studied by Wang et al. [107]. They applied a combination of high- and low-frequency ultrasound treatment to *Scenedesmus dimorphus* and *Nannochloropsis oculate* biomass—and found it to be a promising approach to improve disruption effectiveness, in terms of reduced energy consumption.

Keris-Sen et al. [108] also demonstrated the efficiency of ultrasound upon cell disruption. Upon ultrasonication of mixed microalgal cultures, a 1.5–2-fold increase in extraction of lipids was recorded in the presence of solvents (namely *n*-hexane and chloroform/methanol mixture).

However, their work did not report on the effect of ultrasonic treatment upon changes in microalga cell microstructure, nor did it compare the effect of ultrasonic treatment upon changes in lipid composition.

Although it can be scaled up to an industrial level and operated continuously [90], the energy consumption of ultrasonication is usually above 360 MJ/kg [64]. Additionally, the disruption rate and lipid yield decrease when biomass concentration increases [100,109], which constitutes a challenge to be addressed in further studies. Furthermore, the possibility of forming stable emulsions was also reported [110]. Two major companies are involved in manufacturing ultrasonic devices for industrial-scale applications: REUS (Drap, France) and Hielscher (Oderstraße, Germany). The latter has developed an ultrasonic device with power ranging from 500 to 16,000 W, while REUS builds ultrasonic reactors combined with pumping system, tank draining, and reactor cleaning suitable for reactor capacities between 30 and 1000 L [111].

### 2.3. Bead Milling

Bead milling is a mechanical disruption technique that breaks the cell-wall due to the violent agitation of solid beads against the cells [93,112,113,114]; after the cell disintegration process, the beads can be easily removed from solution by gravity [93]. This technique offers efficient disintegration of wet biomass at different concentrations, as demonstrated by Postma et al. [115]. They investigated mild disintegration of *C. vulgaris* for release of its intracellular compounds—and proved that different stirring speeds (6–12 m/s) could be applied to different biomass concentrations (25–145 g_DW_/kg) to produce over 97% cell disintegration.

Clavijo-Rivera [116] applied bead-milling and high-pressure cell disruption to *P. kessleri* cultures, and demonstrated that both techniques improved lipid recovery. However, bead milling was recommended because it permitted recovery of larger droplets of lipids in supernatants.

Doucha and Lívanský [117] studied the influence of several processing parameters, using different types of homogenizers, to assess the disintegration of wet *Chlorella* cells. Processing parameters included (1) feed rate of *Chlorella* suspension, (2) amount and diameter of beads, (3) agitator speed, and (4) number of passes of microalgal suspension through the chamber. Those authors concluded that an increasing feed rate led to a decrease in the amount of ruptured cells. The opposite happened with increasing agitation speed and number of passes through the chamber. Bead diameters within 0.3–0.5 mm offered the higher efficiency, with breakage yield >90%.

Five cell disruption methods (i.e., direct extraction, sonication, French press, bead-beater, and wet milling) were compared by Shen et al. [118] for their performance upon wet *S. dimorphus* and *C. protothecoides.* Ethanol/hexane and hexane only as solvents were also tested. The authors proved that wet milling, seconded by hexane extraction were the most effective in terms of lipid recovery from *S. dimorphus*.

Moreover, Shen et al. [118] found it possible to enhance bead-milling efficiency by adding solvent(s) to the biomass. They showed that milling, followed by hexane extraction, will increase oil extraction efficiency by 4-fold compared to Soxhlet extraction. Nevertheless, they could not prove the efficiency of this technique for large-scale applications—knowing that it implies higher costs [113], despite its suitability for extraction of value-added compounds [119].

### 2.4. Osmotic Shock

Osmotic shock is a natural process triggered by a sudden change in salt concentration—which will disintegrate the cell wall. Cells are accordingly equilibrated to high osmotic pressure (typically 1 M salt solutions); rapid exposure to low osmotic pressure causes a rapid flux of water into enter the cell—thus building up internal pressure sufficiently to cause cell bursting [120]. The potential of osmotic shock to rupture the cell-wall of microalgae has been recently studied [63,82,121]; when applied, a sudden decrease in osmotic pressure caused cells to rupture [122]. It was previously reported [63] that lipid recovery can be doubled when this technique is applied, relative to extraction via polar and non-polar organic solvents, in the case of *Chlamydomonas reinhardtii* wet biomas*s*. This is a nonexpensive and economically feasible method, yet not all microalgae are broken off through osmotic shock [82,121]; this is indeed a species-dependent process [63]. Even if it is efficient only for a limited number of microalgae, the possibility of its application to some species is desirable—as it will contribute to reduce costs considerably.

### 2.5. Enzymatic Hydrolysis

**Figure 2:**
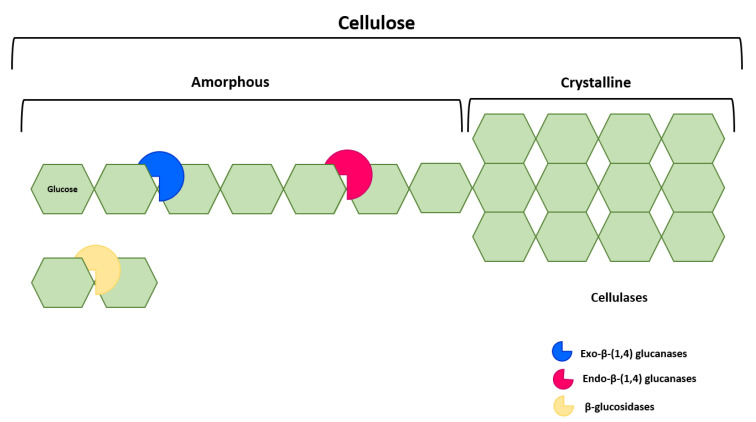
Mechanism of degradation of cellulose via hydrolytic enzymes.

Enzymatic hydrolysis consists of the breakdown of a compound in the presence of an enzyme, following its reaction with water [123]. Enzymatic pretreatment aimed at hydrolyzing microalgal cell walls is a promising, non-destructive procedure—which provides efficient recovery of lipids, and does not damage intracellular compounds for resorting to specific enzymes [122,124]. To design an efficient enzymatic procedure, it is essential to accurately know the cell wall composition—so as to select the most appropriate enzymes and obtain a high extraction yield. Enzymatic pretreatment leads to a significant alteration in the cell wall structure. Cell wall disruption is chiefly caused by cleavage of β-glucosidic bonds in polysaccharides; they are established between the hemiacetal group of a saccharide molecule and the hydroxyl group of some organic group (e.g., alcohol). For instance, in the case of cellulose, hydrolysis is generally brought about by three types of cellulases (Figure 2): endo-glucanases that break the cellulose polymer into smaller chains, thus exposing reducing and nonreducing ends; exo-glucanases that act upon these reducing and nonreducing ends, thus releasing cello-oligosaccharides and cellobiose units; and β-glucosidases that finally cleave cellobiose to free glucose—and thus completing hydrolysis [125].

Zhang et al. [126] tested enzyme-assisted extraction of lipids from *Scenedesmus* sp. fresh biomass using cellulase, xylanase, and pectinase. Their results in terms of recovery of lipids and fatty acid methyl esters (FAMEs) were ca. 2-fold those of the enzyme-free control group. Enzymatic pretreatment led to hydrolysis of polysaccharides into short-chain oligosaccharides and/or monosaccharides. To compare untreated and enzymatically treated *Scenedesmus* sp., the content of the cell wall in main organic carbon groups was analyzed. Results showed that the ratio of bonds C−C/C−H and ether bonds C−O in untreated and enzymatically treated microalgae is 3.33 and 4.56, respectively, thus accounting for ca. 90% of the total organic carbon groups in the cell wall. As described in Section 1.3, the algal cell wall contains a polysaccharide specific matrix and glycoproteins. Reduction of ether groups therein was attributed to hydrolyses of β-glucosidic linkages by enzymes [127]; hence, the absolute content of bonds C−C/C−H remained stable.

Enzymatic hydrolysis using cellulase was tested by Cho et al. [128] upon *C. vulgarism*—who observed an enhancement in lipid extraction by 1.73-fold relative to unhydrolyzed cultures. Snailase and trypsin have been claimed as enzymes able to provide higher yields of oil recovery (ca. 35%) in comparison to cellulase (16%), neutral protease (12%), or alkaline protease (8%) [129]. On the other hand, Cheng et al. [130] obtained lower extraction yields with snailase (7%), again upon *C. vulgaris.*

Enzymatic pretreatment of the sugars present in microalgal biomass offers many advantages over e.g., chemical hydrolysis with acids or alkalis. Since this method does not require expensive equipment, neither does it generate toxic products [72], it appears as a promising approach in attempts to improve lipid extraction from microalgae.

### 2.6. Acid Hydrolysis

Strong acids, such as hydrochloric, sulfuric, or nitric, are commonly used in this pretreatment—even though several factors, such as acid strength, duration of treatment, and temperature, considerably affect the yield of acid hydrolysis, depending on the microalgal species at stake [131].

From a literature search, acid hydrolysis has proven a simple and effective method to extract lipids from wet biomass. It mainly targets release of bound lipids by weakening lipid-starch and -protein intermolecular forces [132] and is therefore required for extraction of otherwise bound lipids. This technique minimizes disturbances caused by the water phase, being more effective than physical or thermal methods; evidence in this regard was provided by Yu et al. [133]. Their results pertaining to different cell disruption methods unfolded distinct amounts of lipid content: Soxhlet (12.8%), HCl digestion (11.2%), bead-beating (6.6%), sonication (4.4%), microwave (0.9%), and autoclaving (0.6%); and showed to be effective to extract all essential fatty acids in the tested microorganisms. In another study, HCl digestion led to appearance of linolenic acid in the fatty acid profiles of *Chlorella sorokiniana*; this compound has been widely used in supplementation of many functional foods [134]. This type of digestion has also been used in many standard lipid extraction methods [132,135].

Sulfuric acid is also commonly utilized at industrial scale to provide highly effective and inexpensive cell disruption [136]. This type of acid hydrolysis was investigated by Duongbia et al. [137], who obtained 17.15 wt% hydrolysis at 100 °C for 60 min, followed by extraction with *n*-hexane for 30 min. Those authors also observed that an increase from 0.5 to 2 M in sulfuric acid concentration led to increased lipid concentration; however further increase to 5.5 M produced a decrease in yield, because excess sulfuric acid degraded lipids themselves. Park et al. [138] studied the efficiency of sulfuric acid–catalyzed hot water extraction of lipids from wet *C. vulgaris*, under 1% and 0.5% (*w*/*w*, at different temperatures and reaction times. At 120 °C for 60 min, the lipid extraction yield was 337.4 mg/g_cell_; at 150 °C for 8 min, 337.5 mg/g_cell_; and at 150 °C for 16 min, 334.2 mg/g_cell_. Between 120 and 150 °C, the lipid extraction yields were comparable, so reaction time can be reduced from 60 to 8 min. Those authors also confirmed that the original lipids in *C. vulgaris* cells were extracted without change in their molecular structure.

In attempts to attain a more efficient process for production of lipids and carbohydrates, Martins et al. [139] proposed lipid extraction using various methods and a multistage strategy—by resorting to a mixture of ethanol and hexane. Dilute sulfuric acid-mediated hydrolysis of *C. vulgaris* was used to disrupt cell walls and improve multistage extraction of lipids—in a carbohydrate-lipid route; lipid yield was twice that following the lipid-carbohydrate alternative route.

Hydrolysis of microalgal biomass using nitric acid was claimed to be more efficient than with other strong acids [140]. According with Lee et al. [141], hydrothermal nitric acid pretreatment can be a promising route to extract microalgal oil; they demonstrated that such a compound is a potent acid catalyst, able to effect extraction of lipids from *Nannochloropsis salina*—and further control their properties. The maximum lipid yield, 24.6%, was obtained with 0.57% nitric acid, upon optimization through response surface methodology.

Acid hydrolysis pretreatment is easy to scale up, as demonstrated by the National Renewable Energy Laboratory (Golden, CO, USA). High efficiency of oil recovery from dilute acid pretreatment was reported by Laurens et al. [142], who were able to release 97% of the total fatty acid residues using hexane. The same authors demonstrated that carbohydrates were also hydrolyzed, thus allowing subsequent bioethanol production as a co-product—which helped reduce overall costs.

Another approach led to recovery of up to 87% of fatty acid residues and 74% of carbohydrates via hexane extraction—the latter readily fermentable for ethanol production; it has been termed Combined Algal Processing (CAP), and was proposed by Dong et al. [64]. This process avoids solid/liquid separations to extract lipids, because the pretreated algal slurry is transferred directly to a fermenter for ethanol production—which will afterward be recovered by distillation, and lipids extracted by hexane. At the same time, putative emulsifiers present do not act because they are simultaneously hydrolyzed. This appears to one of the most promising methods at industrial scale—as it possesses low energy costs, besides being quite efficient in disrupting the microalga cell wall.

### 2.7. Ionic Liquids

The idea of resorting to ionic liquids to assist in lipid extraction from wet microalgae is relatively new. These are green solvents designed to replace toxic organic solvents; they are capable of fusing without decomposing within a range of temperatures that varies between 0 and 140 °C. Chemically speaking, ionic liquids are salts made up of an organic cation and an inorganic anion; they are flexible solvents because of the specific way anion and cation bind—thus allowing them be adapted to the specific needs of the user, in terms of polarity, conductivity, hydrophobicity, and solubility [65].

The potential of ionic liquids to extract wet microalgal biomass has been addressed by Orr et al. [143]; They used 1-ethyl-3-methylimidazolium ethylsulfate [C_2_mim][EtSO_4_], at room temperature, to disrupt *C. vulgaris* in conjunction with methanol. An easy recovery of lipids resulted, along with a large degree of dewatering of the biomass (0−82 wt.% water), within a short time (75 min). These results are auspicious, for implying cell rupture and lipid extraction demanding low energy consumption—besides being compatible with wet biomass.

Similar use was examined by Chen et al. [144], to assist subcritical water in extracting lipids from wet *Scenedesmus* sp.; the ionic liquid used was [HNE_t3_] [HSO_4_], at 110 °C. A typical lipid yield of 35.7% dry weight biomass was obtained; this value is closer to that via Bligh and Dyer’s methodology (i.e., 35.3% dry weight biomass). Comparison between the latter method and ionic liquid [P(CH_2_OH)_4_]Cl toward lipid extraction using wet (71.7% water content) and dry biomass from *Nannochloropsis oculata* was performed by Olkiewicz et al. [145]; remember that Bligh and Dyer’s method is often used as a standard method, owing to the possibility of application to biomass with moisture content up to 80% [146]. Use of wet biomass increased lipid yield from 17.3% to 19.5%, and from 12.8% to 14.6% for Bligh and Dyer’ method and ionic liquid, respectively. Conversely, Young et al. [147] achieved 25% lower extraction efficiency of lipids with wet biomass when compared to dry biomass; the difference may derive from use of a different ionic liquid ([C_2_mim][MeSO_4_]).

Lipid extraction from wet microalgal assisted by ionic liquids decreases lipid contamination, is environment-friendly, and easy to scale up. After having extracted lipids, the recovery of ionic liquids themselves should be addressed—before this method can be eventually scaled up.

### 2.8. Patented Processes

The most commonly used technologies for lipid extraction from microalgae (i.e., organic solvent, Soxhlet, mechanical break) apply mainly to laboratory stage; the associated processes are indeed complicated, the oil extraction rate is low, the cost is high, and the energy consumption is significant—all of which greatly constrain their industrial application. Currently, the dry microalgal powder solvent extraction method is the most widely used; knowing that the water content in microalgae can be as high as 80% (*w*/*w*), one realizes that the energy consumption of drying alone overrides most other extraction costs. Therefore, development of feasible methods from wet biomass are urged—able to easily and economically extract mixed fatty acids from microalgae [37].

In an effort to contribute to the aforementioned development, a list of patents was selected (Table 1) that convey new and innovative methods—bearing a with potential for future implementation at large scale.

Zhanyou et al. [148] provided a new method for extracting oil from wet microalgae, which circulates extracted water to grow microalgae. After concentrating wet algae, an ionic liquid and carbonate are used to break the cell wall, followed by salt extraction with an ethanol-water-sodium carbonate system. The final upper phase consists of a mixture of fatty acids and ethanol, whereas the lower phase contains a large amount of sodium carbonate—suitable for cyclic microalgal culture. The cost, associated with the energy consumption of spray-drying is thus saved; while the residual liquid generated by the process is effectively used, thus raising a lower pressure upon the environment.

In view of the shortcomings discussed above, Jie et al. [149] provided a method for extracting mixed fatty acids from wet microalgae slurry using subcritical water as extraction medium and hydrolysis reagent, thus avoiding the need of organic solvents. Relatively severe subcritical conditions are used to destroy the cell walls of microalgae, thus paving the way for rapid and efficient extraction of oil therefrom.

Although thermal cycle technology for drying microalgae have experienced continuing developments, a comprehensive energy assessment of drying microalgae prior to extracting oil is still lacking. The invention by Chunfeng et al. [150] addresses this particular, based on a microalga treatment device integrated with vapor recompression and heat exchange; it includes three drying systems, oil extraction, and solvent recovery. Compared to the traditional process, energy of the entire process is saved by 35.3%—which corresponds to a decrease in energy consumption 14.55 MW of the traditional process to 9.43 MW of this process.

According Wenzhou et al. [151], no suitable method for large-scale extraction of microalgal oil has been established on a global scale. As safe and effective organic solvent, ethanol is essentially harmless to humans, and can be used effectively to extract oils. Nevertheless, research on use of ethanol to extract oil from wet microalgae, under normal temperature and pressure, is scarce. Therefore, extraction of oil from microalgae using ethanol was studied by those authors—and a simple, safe, effective, and low-cost method was claimed. Proteoglycans are also obtained as side product—which, together with oil, can be used as ingredients of health products, cosmetics, or feed. Microalga biomass is accordingly added to the ethanol solution and stirred to promote mass transfer; the microalga residue is separated by filtration or centrifugation. The upper layer is collected, and the residue is repeatedly extracted with the ethanol solution. The remaining residue is washed and dried, to obtain the crude proteoglycan; while all upper layers are combined and concentrated to obtain crude oil—up to an extraction efficiency of 99%.

The studies discussed in Section 2.2 pointed out at having a good processing effect toward extraction of oil from microalgae. However, the effect of ultrasound upon the structure of cells (and cell walls, in particular) should be known, so as to rationally optimize the corresponding process of oil extraction. Jun et al. [152] accordingly suggested a method to alter the fractal structure of wet microalgae cells, and thus promote the extraction of oil by ultrasonic waves. This method comprises harvesting of wet microalgae with a fractal dimension of 1.21–1.24 and a cell wall thickness of 0.07–0.08 μm, followed by modification via ultrasonic irradiation.

The bottom line is to control the power and time of ultrasonic irradiation so as to increase the cell fractal dimension to 1.46–1.51, and concomitantly reduce their cell wall thickness to 0.04–0.06 μm. The extractant is finally added to the wet microalgae toward oil extraction.

We recall that the most common methods of destroying cell walls include ultrasonic vibrations, low and high temperatures, osmotic shock, and mechanical destruction. According to Aleksandrovich et al. [154], none of these methods guarantees destruction of all microalgae cells membranes, namely of *Chlorella* genus—because (unlike other genera of microalgae) they possess robust cell membranes. Therefore, they proposed a method for higher yields in lipid extraction involving disruption of cell walls in a vortex electromagnetic field with ferromagnetic particles—which apparently led to a fragmentation degree of 99.8%. Subsequently, the biomass is subjected to multiple extractions, carried out in a multistage apparatus using an organic solvent (e.g., chloroform, carbon tetrachloride). Thee extract and defatted biomass are eventually separated by centrifugation. The extract consists of a mixture of tri- and diacylglycerols in the organic solvent; while the skimmed biomass contains a mixture of bark residues, cellular proteins, and minerals—suitable for use as protein additives in feed.

The last invention in Table 1 relates to the technical field of microalgal energy, in particular a method for obtaining purified microalgal oil via ethanol dehydration—wet oil extraction—*n*-hexane extraction. Distinct technical solutions were adopted, namely: (1) dehydration of microalgae wet biomass with an ethanol solution, (2) extraction of oil with a non-toxic organic solvent; (3) addition of water and inorganic salts to the crude oil obtained in step 2), to form an aqueous crude oil solution, addition of *n*-hexane to the aqueous crude oil solution, and then centrifugation—with the top layer containing the oil; an (4) solvent removal from the top layer solution, thus leaving microalga oil behind. The ethanol dehydration treatment of harvested microalgae before extraction of oil reduces indeed the energy consumption required for drying the microalgae, and dramatically reduces the moisture of microalga sludge during the process. In the *n*-hexane extraction process, the removal rate of proteins, pigments and other substances in crude oil is high, the loss of esterified fat is small, and the process does not change the composition of fatty acids.

## 3. Microalgae Integrated Processes

### 3.1. Microalgae-Based Processes

Microalgae exhibit a high potential for use in several sectors, such as food and feed (nutraceuticals and aquaculture nutrients), pharmaceuticals, cosmetics, and chemicals (fine materials), energy (biofuels, power, heat)—besides ecological uses, such as in greenhouse gas mitigation and wastewater treatment [156]. Therefore, small/medium-sized facilities and pilot/demonstration operations have been made available, and the value of microalga-based products has undergone improvement [157]—with a number of associated technologies developed and duly patented [158,159]. Considerable progress was made regarding microalga commercialization, yet several technical and economic difficulties persist.

Microalgal biomass production requires long times and large amounts of culture volumes to be industrially feasible. Additionally, its commercialization is often based upon extraction of a single compound, so co-products are devalued or even excluded. Considering that extraction and purification of compounds require refined techniques, and that these procedures are energy-consuming, integration with other process units should be addressed [159,160]. Such integrating brings advantages toward industrial operation; the underlying synergistic strategy will reduce processing time and improve economic feasibility. Hence, the subsequent sections critically discuss alternative forms of integration.

### 3.2. Process Integration Strategies

Process integration has been carried out in several areas of small- and large-scale importance [161]—although in essence this is an engineering approach directed to design and operation of industrial systems. The main objective of process integration is to take advantage of individual unit operations from a global perspective, thus improving overall efficiency of the production chain; issues related to environmental policy and bioeconomy are also to be addressed [162]. In the last decade, new trends in process integration encompassing microalgae have emerged—likely to achieve greater profitability and environmental sustainability, while producing a wide array of products and energy.

Microalga-based processes may be regarded as integrated, either due to recovery of different compounds from the microalgal biomass or by employing raw materials supplied by adjacent industrial units. For instance, Aziz et al. [163] proposed an energy-efficient drying method based on enhanced process integration; harvested biomass from *Chlorella* sp. was accordingly dried, thus achieving a high calorific value. Hot dried microalgae flown to a gasification stage is then feasible for thermochemical conversion and production of syngas—which is, in turn, used to generate power through combined cycle technology. The products are electricity and a CO_2_-rich flue gas; a small part of the electricity generated is used in the drying stage, and the flue gas is recycled and used to preheat the wet, freshly harvested microalgae in the drying process. The chemical components of the flue gas serve, in turn, as nutrients for microalgal growth. Using this integrated process, the total energy required for microalga utilization is significantly reduced.

Luo et al. [164] proposed a different integration process, and suggested a scheme leading to production of biofuels from microalgae assisted by ultrasounds—along with discussion of the influence of ultrasonic energy in each step of the whole process. Ultrasonic energy can stimulate cell growth since it enhances nutrient assimilation, accelerates cell agglomeration, and improves cell disruption and solvent-mediated extraction. It is also described as a facilitator of transesterification. Ultrasound seems to positively influence pretreatment and thermochemical/biochemical conversion of microalgal biomass. However, integration of ultrasonic intensification with chemical energies, ultrasonic reactor design, and cost of ultrasonic operation and catalysts themselves may hamper its large scale application.

Modeling and simulation have been used as tools to study the integration of microalga-based processes with other biorefineries. Moncada et al. [165] modeled the economic and environmental advantages and disadvantages of a microalgae-based and a sugarcane-based biorefinery consortium. Two different scenarios were considered for the sugarcane: the first one with combined production of sugar, ethanol, and electricity; the other added cultivation of *Chlorella* sp., using CO_2_-rich streams derived from fermentation and cogeneration systems, to the first one. The oil extracted from microalgae was used as raw material to obtain biodiesel and glycerol, as well as electricity, ethanol, and sugar. From environmental and economic perspectives, the second scenario held the best performance—and exhibited a potential to reduce CO_2_ emissions by 39% compared to the first scenario. Using process modeling tools, Moncada et al. [166] similarly studied the integration of castor beans and microalga processing toward production of several value-added products (i.e., polyol, ethylene glycol, omega-3 acid, biodiesel, and methanol). Castor bean cake and microalgal paste were used to feed a Biomass Gasification Combined Cycle Cogeneration system (BIGCC), where part of the CO_2_ produced as flue gas was captured and employed as substrate for microalga growth in a four-stage process: (1) castor oil extraction and polyol production; (2) biomass-fired cogeneration system, where the residues from the extraction of oils are used to feed the BIGCC; (3) microalga growth (*C. vulgaris*), oil extraction and biodiesel production; and (4) ethylene glycol production, with glycerol obtained from the BIGCC process being reacted with hydrogen. Both studies proved the importance of analyzing each type of method from economic, environmental, and social perspectives—beyond the technical feasibility only, from an experimental point of view. The scenario involving the highest integration level produced the best economic and environmental performance. This form of analysis constitutes a step towards efficient development and conceptual assessment of such biorefineries to industrial scale—while breaking barriers related to use of microalgae as potential feedstock to obtain biocompounds and fuels.

Zhu and Hiltunen [167] proposed a framework to enable application of livestock waste compost to cultivate microalgae and produce high-value bioproducts, biodiesel, and biogas—through system integration and engineering. This represents an example of a sustainable way to cultivate microalgae, since livestock waste will always be generated. Among the germane parameters tested, the authors highlighted: geographical variation (changes in weather, temperature, and light intensity affect microalgae growth); light penetration; livestock waste type (since different types of slurry show variability in their nutrient contents); competitive microorganisms (as bacteria might appear and competitively affect growth of microalgae); microalga growth characteristics (it is crucial to select a species capable of growing under the proposed conditions); photosynthetic efficiency of microalgae (sensitive to weather conditions); and construction and operating costs (including energy input, cleaning, sterilization, maintenance, and chemicals consumption). Further investigation concerning growth of microalgal biomass in livestock waste compost is still required; however, integration with livestock waste compost already proved useful for bioproduct production and waste management.

Chen et al. [168] suggested development of a system that integrates sludge digestion with microalga cultivation, to convert effluent wastes to biogas and biochemicals. Recall that wastewater treatment plants (WWTPs) generate a tremendous amount of waste activated sludge (WAS); treatment of WAS is accomplished via anaerobic digestion (AD), to convert existing organic waste to bioenergy. Since WAS has a complex composition, the efficiency of biogas production is limited [169,170]; it contains 20–60% CO_2_ and 0.005–2% H_2_S, thus making it unfavorable for use as a fuel gas without further purification [171,172]. As microalgae can rapidly mitigate the CO_2_ and H_2_S produced during the process, this considerably improves biogas quality; hence, they are regarded as a possibility to convert effluent wastes to biofuels and biochemicals. Biogas/volatile fatty acids (VFAs) (the main byproduct of anaerobic fermentation) can significantly limit biogas production, but it can be efficiently converted to acetyl-CoA (the main precursor for lipid synthesis) using microalgae.

After effluent treatment and biogas upgrading using microalgae, the sludge and microalgal residues should be treated to minimize the risk of pollution; they may accordingly be converted to biochar (i.e., a carbonaceous material suitable as either as a solid-fuel or as a porous carbon material for removing various inorganic and organic pollutants) through thermal conversion. The pyrolysis process has a positive net energy production, while hydrothermal treatment can be chosen as the wet biomass conversion method. Additionally, this study provided useful information for combining sludge digestion with microalgal cultivation—and thus reduce pollutant levels, while simultaneously producing bioenergy.

Colling Klein et al. [70] studied microalgae as a source of biofuels and other bioproducts in the near- to medium-term, by coupling production with large-scale facilities aiming at process integration. Those authors analyzed the main inputs of microalga cultivation and how integrated biorefineries could supply them—giving a special focus to Brazilian sugarcane mills. Those industrial plants are able to supply cheap carbon for microalgal growth in the form of CO_2_ from boiler emissions, ethanol fermentation off-gas, or biogas from vinasse anaerobic digestion; water, organic molecules, and nutrients from natural or processed vinasse; and renewable electrical energy obtained from sugarcane bagasse and straw burning. Many of these companies still lack large-scale facilities, either for cultivation or microalga post-processing. Microalga companies could benefit from the existing infrastructure of sugarcane mills in Brazil, to establish pilot plants and industrial-scale units—and thus stimulate development of this technology for biomass production. The focus should be given to utilization of CO_2_ produced during ethanol fermentation, and CO_2_ contained in the biogas obtained from anaerobic digestion of vinasse to maximize practical integration instead of utilizing flue gas generated—which would require extensive processing before injection in cultivation systems. Scientific data concerning growth of different microalgae species within natural and digested vinasse are still needed, as well as innovative configurations for biogas upgrading—with microalgal cultivation systems and novel bioreactor designs aimed at optimizing CO_2_ uptake, as well as microalga biomass concentration and productivity.

### 3.3. Environmental and Economic Considerations

Integration of processes aims also at reducing greenhouse gas emissions, remediation of effluents, and use of added value co-products—so as to become economically feasible and environmentally sustainable, as per the point of view of a circular biorefinery. This calls for efficient recycling of products generated in the integrated system; wastewater and exhaust gases should indeed still serve as nutrients for growing microalgal biomass, thus also generating a revenue for the industry.

## 4. Concluding Remarks

Disruption of microalgal cell walls and recovery of intracellular lipids starting exclusively from wet biomass are relevant to the economic feasibility of microalga-based biofuel and biochemical production, but they add to the complexity of the underlying process; various possibilities have been proposed to date, and were duly discussed above.

Pretreatment methodologies applicable to wet biomass should be environment-friendly, inexpensive, selective, smooth, controllable and universal. Mechanical methods, such as bead milling and ultrasound, or electromagnetic methods, such as microwaves, seem appropriate—because they are easy to scale-up and do not contaminate the final biomass. However, improvements are still necessary to reduce the associated energy toll. Using a higher biomass concentration, or combining a solvent with other methods can enhance efficiency of extraction—as they use of solvent and energy consumption are reduced; remember that methods resorting exclusively to organic solvents are not environmentally sustainable, and should thus be phased out in the near future. Non-mechanical methods have lower energy requirements and allow a higher quality be reached for the extracted compounds—besides bringing about a more uniform cellular rupture. However, the corresponding treatments are longer, and side products can form due to the high operating temperatures; the process itself is also more challenging from the point of view of control than mechanical methods.

Extensive upgrading and technological advancements have been tested in attempts to directly extract wet microalga biomass, but energy efficiency and cost-effectiveness of cell rupture remain issues to be addressed toward extensive commercialization of microalgal bioproducts; therefore, further research is needed in these areas, aiming at higher disruption efficiencies. New and improved processes are should encompass continuous operation, faster reaction, and lower dependence on water content (or other impurities, for that matter).

Integration of microalga-based processes, under the whole biorefinery concept, is prone to more sustainable and economical processing. However, optimization and scaling up remain as practical challenges; these issues constitute opportunities for R&D efforts in the near future.

## Figures and Tables

**Table 1:** Selected patents related to extraction of lipids from wet biomass.

Patent Name/Invention	Pretreatment Applied to Extract Lipids	Technology to Separate Microalgae Residues	Advantages	Year	Reference
Method for extracting microalgal oil assisted by carbonate and absorbing carbon dioxide in circulated culture	(1) Wall breaking treatment—sodium carbonate + ionic liquid (DBU) + heat (2) Salting-out extraction—methanol (3) CO_2_ introduction (into the aqueous phase)	N/A	Reduces energy consumption cost; Allows water recycling.	2018	[148]
Method for extracting mixed fatty acids from wet microalgae mud by using subcritical water	Subcritical water extraction	N/A	Fast and efficient oil extraction; Environmentally friendly; Uses water as extraction medium and hydrolysis reaction agent (thus avoiding use of organic solvents); Saves energy; High fatty acid extraction rate, simple separation, easy scale-up.	2018	[149]
Joint treatment device based on the integration of vapor recompression and heat exchange and applied to microalgae	Joint treatment device which comprises (a) microalgae drying system; (b) grease extraction system; (c) solvent recovery system.	N/A	Uses dry and wet microalgae; Energy-efficient; Joint device has simple structure and is easy to perform; The whole process reduces operating costs.	2017	[150]
Method for preparing microalgae oil and protein-polysaccharide by adopting wet algae and one-step method	Extraction with solvents–ethyl alcohol	Filtration method, and standing layering or centrifugal layering	Strong fat solubility of ethyl alcohol; Pollution-free to human body and environment; Keeps activity of high value-added active substances; Short treatment time; Simple to operate; High extraction efficiency; Possibility of application to large-scale.	2017	[151]
Method of using ultrasonic wave to change wet alga cell fractal structure for improving grease extraction	Solvent extraction (chloroform-methanol), with previous treatment by ultrasound waves	Centrifugation	Avoids dewatering and drying steps; Reduces cell wall thickness.	2015	[152]
Process for extraction of lipids from microalgae using ionic liquids	Several ionic liquids, individually and in combination, were tested	Chemical forces, magnetic forces, and separation based on density differences were tested	Green technology.	2011	[153]
Method of extraction of lipids from biomass	Cell membranes destruction in vortex-electromagnetic field with ferromagnetic particles	Organic solvent superposition pulse-cavitation	Reduces extraction time; Increases output of lipid fraction.	2015	[154]
Method for wet extraction of purified microalgal oil	Solvent extraction (hexane)	Centrifugation	Easy operation; Low power consumption.	2014	[155]

N/A—Not Applicable.
